# Tumor Necrosis Factor Improves Vascularization in Osteogenic Grafts Engineered with Human Adipose-Derived Stem/Stromal Cells

**DOI:** 10.1371/journal.pone.0107199

**Published:** 2014-09-23

**Authors:** Daphne L. Hutton, Renu Kondragunta, Erika M. Moore, Ben P. Hung, Xiaofeng Jia, Warren L. Grayson

**Affiliations:** 1 Department of Biomedical Engineering, Johns Hopkins University School of Medicine, Baltimore, Maryland, United States of America; 2 Translational Tissue Engineering Center, Johns Hopkins University School of Medicine, Baltimore, Maryland, United States of America; 3 Department of Material Sciences and Engineering, Johns Hopkins University School of Engineering, Baltimore, Maryland, United States of America; University of Pittsburgh, United States of America

## Abstract

The innate immune response following bone injury plays an important role in promoting cellular recruitment, revascularization, and other repair mechanisms. Tumor necrosis factor-α (TNF) is a prominent pro-inflammatory cytokine in this cascade, and has been previously shown to improve bone formation and angiogenesis in a dose- and timing-dependent manner. This ability to positively impact both osteogenesis and vascular growth may benefit bone tissue engineering, as vasculature is essential to maintaining cell viability in large grafts after implantation. Here, we investigated the effects of exogenous TNF on the induction of adipose-derived stem/stromal cells (ASCs) to engineer pre-vascularized osteogenic tissue *in vitro* with respect to dose, timing, and co-stimulation with other inflammatory mediators. We found that acute (2-day), low-dose exposure to TNF promoted vascularization, whereas higher doses and continuous exposure inhibited vascular growth. Co-stimulation with platelet-derived growth factor (PDGF), another key factor released following bone injury, increased vascular network formation synergistically with TNF. ASC-seeded grafts were then cultured within polycaprolactone-fibrin composite scaffolds and implanted in nude rats for 2 weeks, resulting in further tissue maturation and increased angiogenic ingrowth in TNF-treated grafts. VEGF-A expression levels were significantly higher in TNF-treated grafts immediately prior to implantation, indicating a long-term pro-angiogenic effect. These findings demonstrate that TNF has the potential to promote vasculogenesis in engineered osteogenic grafts both *in vitro* and *in vivo*. Thus, modulation and/or recapitulation of the immune response following bone injury may be a beneficial strategy for bone tissue engineering.

## Introduction

Cell-based approaches to bone tissue engineering provide a tremendous opportunity to repair large, non-healing bone defects by enriching the site with regenerative cells. However, the potential benefit is hampered by the need for rapid vascularization to maintain cell viability and also provide complex signaling cues between vasculature, infiltrating inflammatory cells, and osteoprogenitors that guide tissue repair and maturation. Therefore, coupling vascularization strategies with bone tissue engineering may greatly improve functional outcomes.

Previously, our group has demonstrated the ability of low-passage adipose-derived stem/stromal cells (ASCs) to form robust vascular networks within osteogenic tissues *in vitro* with the help of biomimetic spatiotemporal cues [1]. Aggregation of ASCs into multicellular spheroids substantially improved their ability to form interconnected vascular networks. This vascular growth was inhibited in the presence of osteogenic factors, and necessitated the development of a step-wise protocol in which vascular networks were established before osteogenic induction. Furthermore, this composite tissue induction was coupled by platelet-derived growth factor (PDGF), which significantly increased both vessel density and mineralization when added exogenously. PDGF is a key regenerative cue that is released by activated platelets following bone injury [2], raising the question of how other elements of normal bone repair may affect bone tissue engineering.

Immediately following bone injury, there is an acute inflammatory phase in which activated platelets and macrophages release a host of factors, including PDGF and pro-inflammatory cytokines [2]–[5]. These factors play a critical role in the initiation of healing through the recruitment and activation of regenerative cells, as well as promoting re-vascularization. While normally thought of as intrinsically damaging to tissue repair, pro-inflammatory cytokines such as tumor necrosis factor-α (TNF) have been shown to promote tissue-healing processes in some cases. For example, exogenously applied TNF has been shown to promote angiogenesis *in vivo* [6], [7] and *in vitro* by inducing the endothelial tip cell phenotype [8]. This response appears to be highly sensitive to the timing and dosage, as there are also many reports of the anti-angiogenic effects of TNF [8]–[11]. In the case of bone, TNF has been shown to improve bone fracture healing *in vivo* [12], [13] and osteogenic differentiation of stem cells *in vitro* [14]–[17].

The current study aims to understand whether TNF may benefit the development and maintenance of vascular networks within engineered osteogenic tissue. In particular, we study the effects of TNF dose and timing, as well as its combined effects with PDGF. In addition, we generate osteogenic grafts within composite scaffolds to study tissue maturation and integration *in vivo*. We demonstrate that recapitulating the biochemical environment within normal bone healing cascades through the inclusion of the inflammatory mediator TNF improves vascularization of tissue engineered osteogenic grafts.

## Materials and Methods

### Ethics Statement

Human subcutaneous adipose tissue was obtained in the form of lipoaspirate from female donors (n = 3) with written informed consent under the approval of the Johns Hopkins Medicine Institutional Review Board. All animal procedures were conducted in strict accordance with the Guide for the Care and Use of Laboratory Animals by the National Institutes of Health, with every effort taken to minimize animal suffering. The study protocol was approved by the Johns Hopkins University Animal Care and Use Committee. Johns Hopkins University is accredited by the Association for Assessment and Accreditation of Laboratory Animal Care International and holds Public Health Service Animal Welfare Assurance Number A-3272.

### ASC isolation and culture

ASCs were isolated from lipoaspirate tissue as previously described [18]. Briefly, tissue was digested with collagenase (1 mg/mL; Worthington Biochemical Corp.) to isolate the stromal vascular fraction of cells. These cells were plated onto tissue culture plastic, and were termed “passage 0 ASC” when they reached 80–90% confluence. ASCs were used at passage 2 for all experiments. Growth medium consisted of: high-glucose DMEM (Gibco Invitrogen) with 10% fetal bovine serum (FBS; Atlanta Biologicals), 1% penicillin/streptomycin (Gibco Invitrogen), and 1 ng/mL basic fibroblast growth factor (FGF-2; PeproTech). Experimental results were verified by repeating studies with cells from three separate donors.

### Flow cytometry

Passage 2 ASCs were assessed via flow cytometry for surface expression of mesenchymal (CD73, CD105, PDGFR-β) and endothelial markers (CD31, CD34). Briefly, cells were suspended in phosphate buffered saline (PBS) containing 2% FBS and incubated with monoclonal antibodies for 30 min at 4°C. Cells were analyzed with a BD Accuri C6 flow cytometer. Antibodies were purchased from Santa Cruz Biotech (PDGFR-β) and BD Biosciences (all others).

### Cell aggregation via suspension culture

Cells were trypsinized and resuspended at a concentration of 200,000 cells/mL in growth medium containing 0.24% (w/v) methylcellulose (Sigma). The cell suspension was pipetted into 10-cm petri dishes coated with 2% (w/v) agarose to minimize cellular adherence to the dish. After overnight suspension culture, cellular aggregates (**Fig. S1**) were collected with a pipet, and then centrifuged before encapsulation procedures.

### Cell encapsulation in fibrin gels

Cells were suspended in fibrinogen (8 mg/mL final; Sigma) and thrombin (2 U/mL final; Sigma) at a final cell concentration of 5000 cells/µL. Fibrin gels were formed by pipetting 35 µL of gel solution into 6-mm diameter wells and incubating at 37°C for 30 min to allow complete gelation before the addition of culture medium. Samples were cultured for 2 to 3 weeks, depending on the experiment, with medium changed every other day.

### PCL scaffold fabrication and seeding

Polycaprolactone (PCL) was utilized as the base scaffold material for these studies to allow handling and implantation of grafts, as well as its biocompatibility, mechanical properties, and *in vivo* stability [19], [20]. PCL scaffolds were produced as previously described [21]. Briefly, a Syil X4 CNC mill (Syil America) was converted into a custom-built 3D printer with a hot melt pressure extruder attached to the spindle. PCL pellets (Polysciences Inc., Warrington, PA) were melted to 80°C and extruded at a linear speed of 2.7 mm/sec through a 460-µm nozzle. Scaffold sheets were printed with a 0/90° lay-down pattern and 40% infill density. Cylindrical scaffolds were punched from these sheets with dimensions of 4 mm diameter ×2 mm height (**Fig. S2**). Scaffolds were treated with 3 M sodium hydroxide for 1 hour to increase hydrophilicity, followed by 1 hour of 70% ethanol to sterilize. Before cell seeding, rinsed scaffolds were incubated with growth medium at 37°C for 1 hour, then blotted on sterile Kimwipes immediately prior to seeding. Cells were seeded into the scaffold pore spaces with fibrin gel to facilitate vascular assembly and rapid encapsulation of cells. Briefly, 20 µL of fibrin gel solution containing 6×10^5^ cells was pipetted into scaffold pore spaces and allowed to solidify at 37°C for 30 minutes.

### Media preparation

Vascular Medium (VM) consisted of endothelial basal medium-2 (Lonza), 6% FBS, 1% penicillin/streptomycin, 10 ng/mL vascular endothelial growth factor-165 (VEGF; PeproTech), 1 ng/ml FGF-2, and 1 µg/mL L-ascorbic acid-2-phosphate (Sigma). For monolayer osteogenic differentiation experiments, Control Medium consisted of low glucose DMEM (GIBCO Invitrogen), 6% FBS, and 1% penicillin/streptomycin; Osteogenic Medium (OM) consisted of Control Medium plus 10 mM β-glycerophosphate (Sigma) and 50 µM L-ascorbic acid-2-phosphate. To support both vascular growth and osteogenic differentiation, OM was further supplemented with 10 /ml VEGF and 1 ng/ml FGF-2 for studies within fibrin gels and PCL scaffolds.

### Osteogenic and vascular induction

For monolayer experiments, ASCs were seeded at 5000 cells/cm^2^ and cultured in either Control Medium or OM for 21 days. Mineralization was assessed via Alizarin Red S staining, as well as quantification of calcium and DNA content, as previously described [1]. For fibrin gel and scaffold experiments, vascular morphogenesis was induced by culturing cells in VM for up to 14 days. Dual (vascular and osteogenic) induction followed a previously described protocol [1], where samples were cultured in VM for 8 days followed by 13 days of OM supplemented with VEGF and FGF-2. Vascular growth was assessed via immunostaining of fibrin gels (whole-mount) and PCL scaffolds (cryosections). Osteogenic differentiation in 3D samples was also assessed via immunostaining, as well as quantification of calcium content.

### Treatment with TNF and PDGF-BB

**Figure 1:**
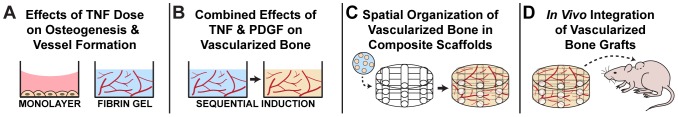
Schematic of experimental approaches. (**A**) Osteogenic (monolayer) and vascular (aggregates in fibrin gel) cultures were studied separately in this experiment to study the effects of TNF dose (0 to 100 ng/ml for the first 2 days only) on each lineage. (**B**) Fibrin-encapsulated ASC aggregates underwent dual (vascular and osteogenic) induction using a step-wise approach [1]. The cells were treated with TNF and/or PDGF-BB to study their individual and combined effects. (**C**) ASC aggregates were seeded with fibrin gel into the pores of 3D-printed PCL scaffolds and induced to form vessels and mineral in order to study the spatial organization of tissue assembly within grafts. (**D**) These cultured grafts were implanted subcutaneously in athymic nude rats for 2 weeks to assess *in vivo* integration and maturation of the grafts.

Different experimental models were employed in this study (**Fig. 1**). Initial experiments tested the effects of acute TNF exposure on osteogenesis and vascularization independently (**Fig. 1A**). ASCs were cultured in either OM (monolayer) or VM (fibrin gel), with the addition of TNF (0, 0.1, 1, 10, or 100 ng/mL) for the first 48 hours to mimic an acute inflammatory response. In the next experiment, the combined effects of TNF and PDGF-BB on dual (vascular and osteogenic) induction were assessed (**Fig. 1B**), as both factors are key mediators in the early bone-healing environment. Fibrin-encapsulated ASCs were treated with 0 or 20 ng/ml PDGF-BB (for the full 21 days of culture) and 0 or 0.1 ng/ml TNF (for 2 or 21 days). To assess tissue development within implantable grafts, ASCs were seeded into PCL scaffolds and underwent vascular and osteogenic induction with the addition of 0 or 0.1 ng/ml TNF (for 2 days) and 20 ng/ml PDGF-BB (for 21 days), and either assessed at the end of *in vitro* culture (**Fig. 1C**) or implanted *in vivo* to assess the survival and integration of the grafts (**Fig. 1D**).

### *In vivo* implantation

Male athymic nude rats (7 weeks old, n = 4 per group; Charles River Laboratories) were anesthetized with isofluorane. Two small incisions were made in the dorsal region of the skin to form bilateral subcutaneous pockets. One sample from each treatment group (± TNF) was placed in each rat (one sample per pocket), then the skin was sutured closed. All rats were sacrificed after 14 days for retrieval of scaffold implants, which were fixed in 3.7% formaldehyde at 4°C for 24 hours before histological analysis.

### Immunostaining and histology

Fibrin gel samples were fixed in 3.7% formaldehyde at 4°C for 3 hours, followed by whole-mount immunostaining, which was performed as previously described [1]. Gels were mounted on glass slides and imaged using a Zeiss LSM 510 confocal microscope with a 5x objective. Fixed PCL scaffold samples were infiltrated with 30% sucrose, frozen in Tissue Tek OCT medium, and cut into 10 µm-thick sections. Cryosections were mounted and dried on Superfrost Plus slides, followed by rehydration in water before staining with either Hematoxylin and Eosin (H&E; Sigma) or immunohistochemistry. Immunohistochemistry was performed by blocking for 30 minutes (10% normal serum/0.2% Triton X), followed by overnight incubation with primary antibodies at 4°C, 1-hour incubation with secondary antibodies at room temperature, and nuclear counterstain for 4′-6-diamidino-2-phenylindole (DAPI; Sigma). Cryosections were imaged using an inverted Zeiss Axio Observer microscope.

Primary antibodies included: mouse anti-human CD31 (4 µg/mL, Sigma and Dako), Cy3-conjugated mouse anti-alpha smooth muscle actin (αSMA; 7 µg/ml, Sigma), rabbit anti-laminin (7 µg/ml, Sigma), rabbit anti-osteocalcin (OCN; 10 µg/ml, Santa Cruz Biotechnology), mouse anti-collagen I (1/1000, Abcam), goat anti-RUNX2 (4 µg/mL, Santa Cruz Biotechnology), and mouse anti-human Lamin A/C (0.5 µg/mL, Abcam). Secondary antibodies (Jackson ImmunoResearch) included: goat anti-mouse (DyLight 488) and goat anti-rabbit (DyLight 649) for *in vitro* samples; donkey anti-goat (AlexaFluor 488), donkey anti-mouse (Cy3), and donkey anti-rabbit (AlexaFluor 647) for *in vivo* samples.

### Image analysis

Confocal z-stacks of immunostained gels were z-projected and thresholded for quantification of vessel network parameters (6 images per group). AngioQuant software [22] was used to quantify total vessel length and number of junctions (“interconnectivity”). Cryosectioned scaffold samples were analyzed with Image J software (NIH). A minimum of 3 slices (spaced ≥100 µm apart) was analyzed from each independent sample. Nuclear counts (DAPI, RUNX-2, Lamin A/C) were performed with the Analyze Particles command on thresholded images and were normalized to the total graft area. Extracellular matrix deposition (“intensity”) was measured by taking the mean grey value of selected regions in non-thresholded images (entire graft region for Col-1 and OCN, and CD31+ overlay region for *in vitro* laminin). Percent vessel area (either CD31 for human vessels or laminin for rat and human vessels *in vivo*) was determined in cryosectioned samples by measuring the thresholded vessel area divided by the total graft area (mm^2^/mm^2^).

### Quantitative RT-PCR

Total RNA was isolated using a TRIzol (Invitrogen) extraction method and quantified using a NanoDrop spectrophotometer (Thermo Scientific). Reverse transcription was performed with 1 µg of total RNA using iScript cDNA Synthesis Kit (BioRAD). Complementary DNA was amplified using SYBR Green PCR Master Mix (Applied Biosystems) and a StepOnePlus Real-Time PCR System (Applied Biosystems). The primer sequences used for PCR analysis are listed in **Table S1**. Expression levels were calculated by the comparative C_T_ method using GAPDH as an endogenous reference gene.

### Statistical analysis

Statistical analyses were performed using GraphPad Prism 5 software. Quantitative data are expressed as mean ± standard error. An unpaired *t*-test was used for two-group comparisons. Multi-group comparisons were determined by either one-way ANOVA with Tukey's post-test (for single factor studies) or two-way ANOVA with Bonferroni's post-test (for multiple factor studies). Significance is denoted as *p<0.05, **p<0.01, ***p<0.001.

## Results

### Cell characterization

**Figure 2:**
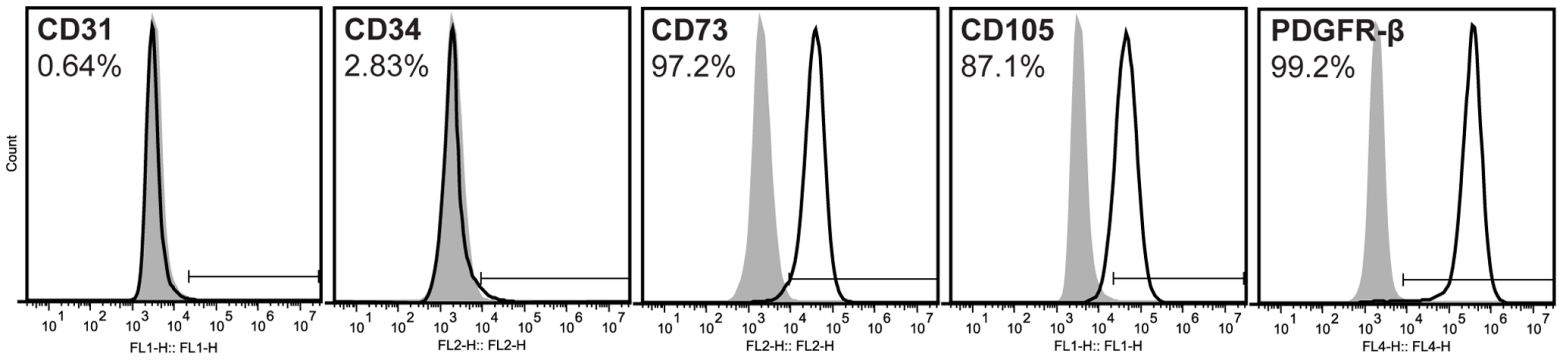
Surface marker characterization of cell population. Flow cytometry histograms from a representative population of passage 2 human ASCs for mesenchymal markers CD73 and CD105, pericyte marker PDGFR-β, and endothelial markers CD31 and CD34. Grey histogram: cells labeled with isotype control antibody; black outline histogram: cells labeled with antigen-specific antibody.

ASCs at passage 2 were predominantly positive for mesenchymal markers (97.2% CD73+, 87.1% CD105+, and 99.2% PDGFR-β+), with very few cells positive for endothelial markers (0.64% CD31+ and 2.83% CD34+) (**Fig. 2**). All CD31+ cells were also positive for PDGFR-β+, indicating that these cells are capable of detecting PDGF-BB directly (**Fig. S3**).

### Effects of acute TNF exposure on independent lineage induction

**Figure 3:**
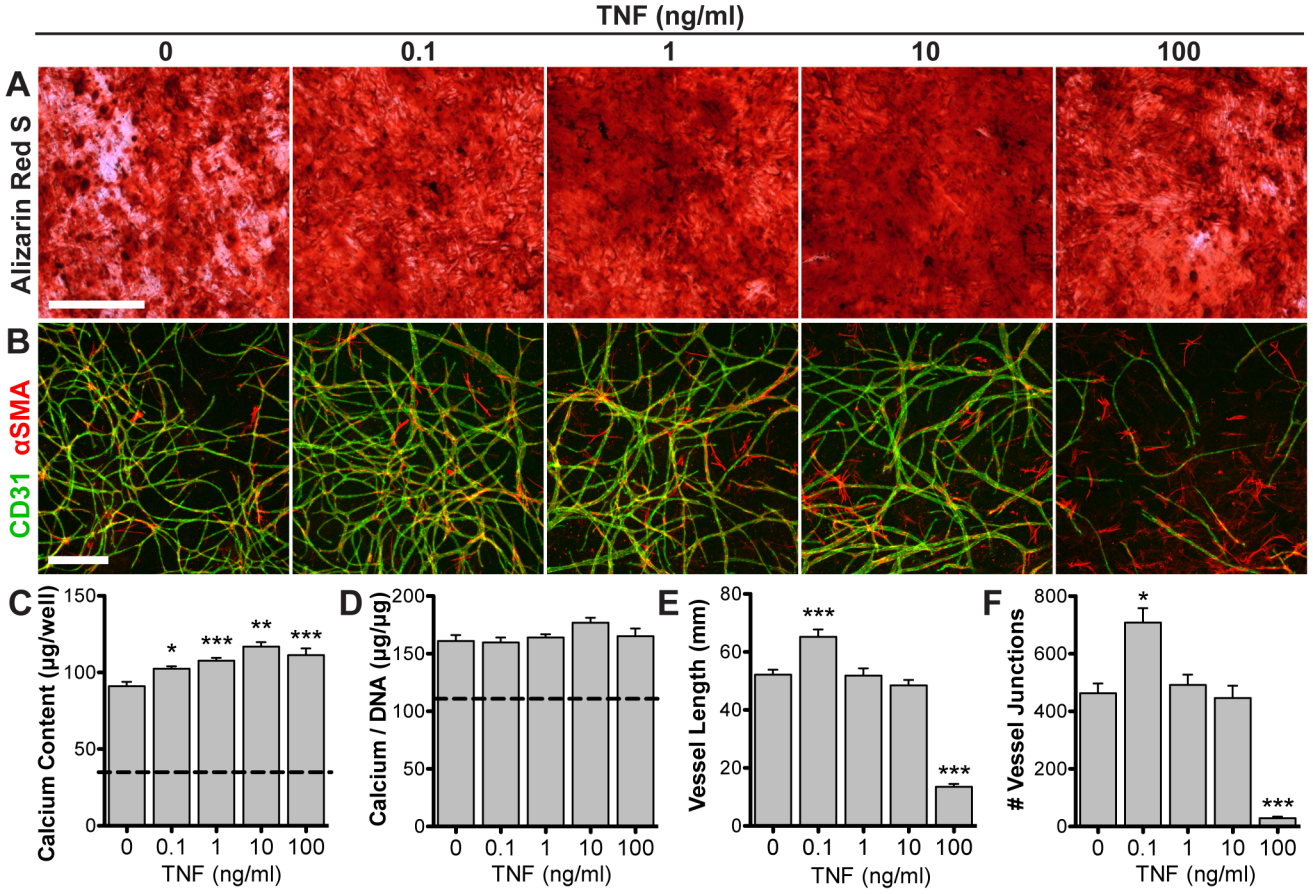
Effects of acute TNF exposure on independent lineage induction. ASCs were induced towards either osteogenic differentiation (2D monolayer) or vascular morphogenesis (spheroids in 3D fibrin gel) and treated with varying doses of exogenous TNF for the first 48 hours. Osteogenic cultures were assessed via Alizarin Red S stain for calcium deposits (**A**), as well as quantification of total calcium content (**C**) and calcium normalized to DNA content (**D**) (dotted line: non-osteogenic control). Vascular cultures were assessed via whole-mount immunostaining for CD31 (green) and αSMA (red) (**B**), as well as quantification of vascular network length (**E**) and interconnectivity (**F**). Scale bars  = 500 µm. Values shown as mean ± SEM. *p<0.05, **p<0.01, or ***p<0.001 versus 0 ng/ml TNF.

The addition of TNF to osteogenic cultures resulted in significantly greater calcium deposition, as shown by Alizarin Red S staining (**Fig. 3A**) and quantification of calcium content (**Fig. 3C**). This improved response was highest at 10 ng/mL. Calcium content normalized to DNA content (to account for differences in cell number) shows no significant difference due to TNF (**Fig. 3D**). In vascular cultures (**Fig. 3B**), 0.1 ng/mL TNF significantly increased vascular network length (**Fig. 3E**) and interconnectivity (**Fig. 3F**). Higher doses showed no change (1 and 10 ng/mL) or significantly inhibited vascular growth (100 ng/mL).

### Combined effects of TNF and PDGF-BB on dual induction

**Figure 4:**
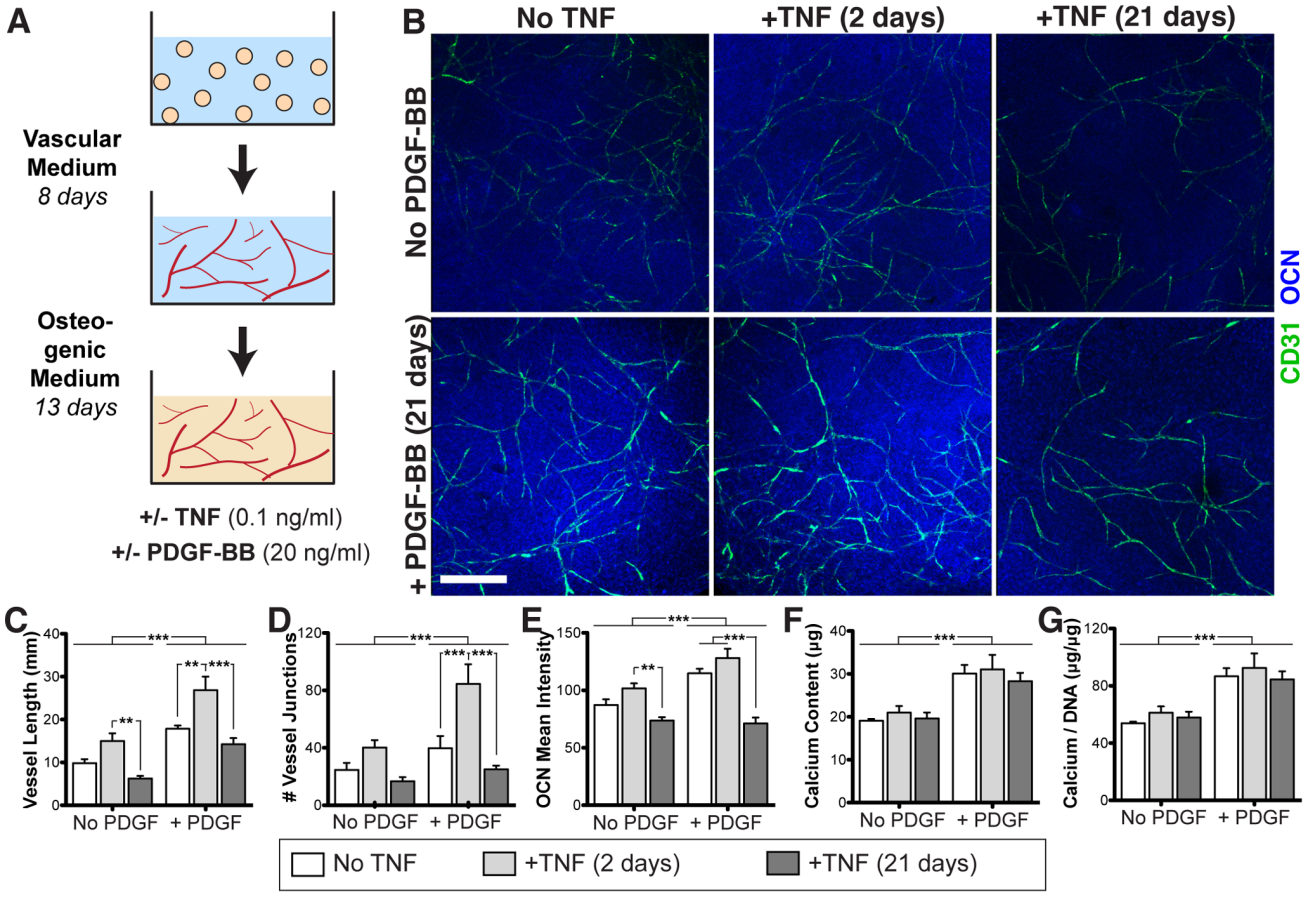
Combined effects of TNF and PDGF-BB on vascular and osteogenic induction within fibrin gels. (**A**) Fibrin-encapsulated ASC aggregates underwent dual induction with the addition of TNF and/or PDGF-BB. (**B**) Whole-mount immunostaining for CD31 (green) and OCN (blue). Scale bar  = 500 µm. Quantification of immunostains: (**C**) vascular network length, (**D**) vascular network interconnectivity, and (**E**) mean intensity of OCN deposits. (**F**) Total calcium content. (**G**) Calcium content normalized to DNA content. Values shown as mean ± SEM. Significance indicated as **p<0.01 or ***p<0.001.

Fibrin-encapsulated cell aggregates underwent vascular and osteogenic induction according to a previously established step-wise protocol (**Fig. 4A**) [1]. Vascular growth was enhanced after acute (2 days) TNF exposure (**Fig. 4B–D**), similar to the vascular-only cultures (**Fig. 3B**). However, chronic (21 days) exposure to the same concentration of TNF significantly reduced vascular growth, and was slightly inhibitory as compared to no TNF treatment. PDGF-BB significantly increased vascular growth in all groups, and demonstrated a synergistic increase in vessel interconnectivity in combination with TNF (interaction p-value  = 0.0496). PDGF-BB also induced a significantly increase in osteogenesis (**Fig. 4E–G**), whereas TNF exposure did not elicit any significant differences, except for a reduction in osteocalcin staining with chronic (21 day) TNF exposure.

### Tissue assembly in composite scaffolds

**Figure 5:**
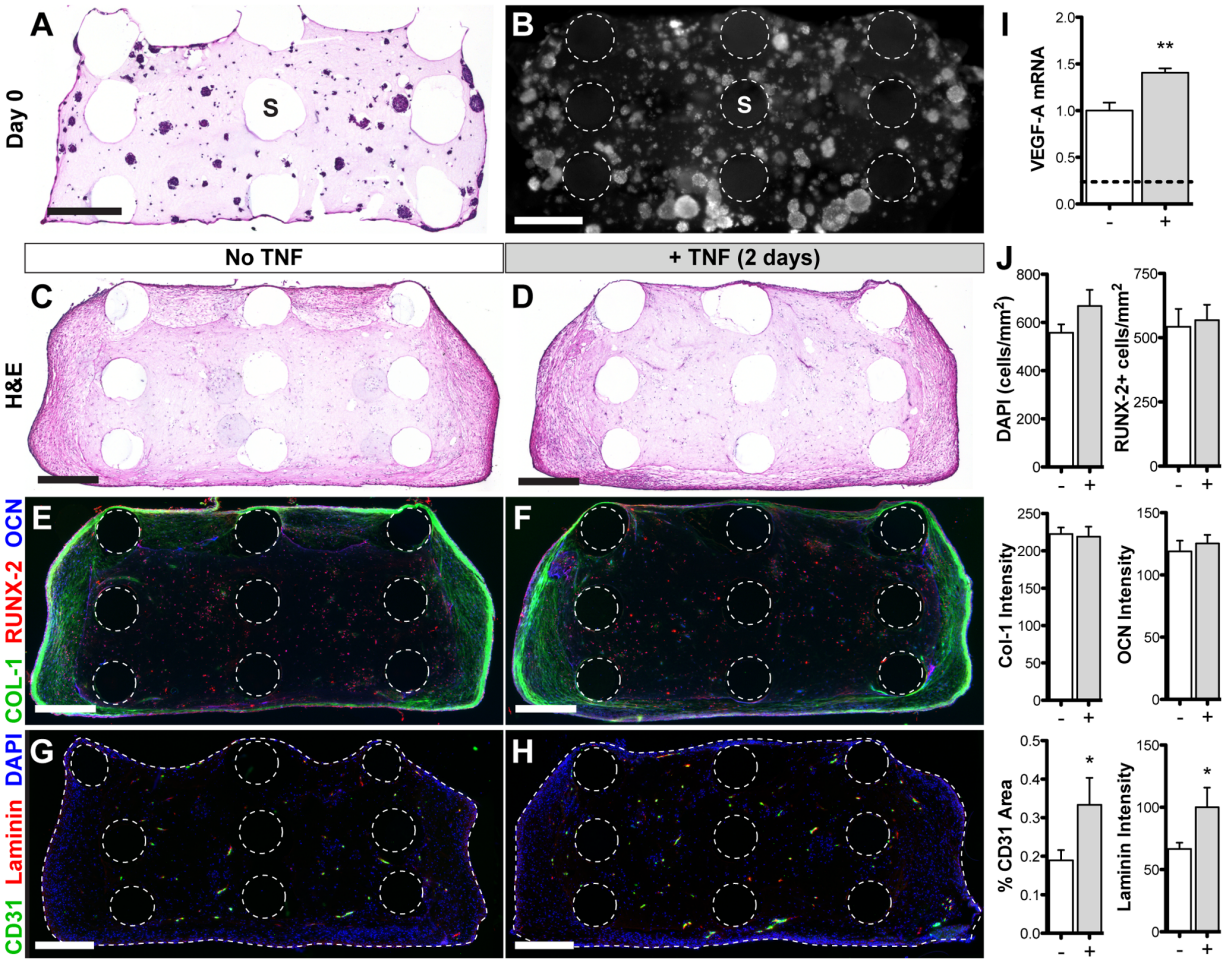
Tissue assembly within PCL-fibrin composite scaffolds. ASC aggregates were suspended in fibrin solution and seeded into the open pores of PCL scaffolds for gelation. Initial cell distribution is shown via H&E staining of a 10 µm-thick section (**A**) and a cross-sectional view of whole-mount DAPI staining (**B**) (PCL scaffold rods: ‘S’). Samples were induced to form vessels and mineral for 21 days in the presence of 20 ng/ml PDGF-BB and either no TNF (**C, E, G**) or 0.1 ng/ml TNF for 48 hours (**D, F, H**). Cryosections (10 µm thick) were stained with H&E (**C, D**), as well as immunostaining for osteogenic (**E, F**) and vascular (**G, H**) markers. Dotted lines indicate the scaffold rods and tissue boundary (**E–H**). Scale bars  = 500 µm. (**I**) VEGF-A relative gene expression (dotted line indicates day 0). (**J**) Quantification of immunostaining. Values shown as mean ± SEM. Significance indicated as *p<0.05 or **p<0.01.

ASC aggregates seeded with fibrin gel into PCL scaffolds were evenly distributed throughout the pores at day 0 (**Fig. 5A, B**). After 21 days of vascular and osteogenic induction, new tissue formed more densely around the periphery of the scaffold, with no noticeable differences amongst treatment groups (**Fig. 5C, D**). Immunostaining for bone matrix proteins collagen I and osteocalcin was denser around the periphery of the scaffold, whereas the majority of cells that stained positively for RUNX-2 (an early osteogenic transcription factor) were located in the central region (**Fig. 5E, F**). Quantification of each of these osteogenic markers showed no statistical change as a result of TNF treatment (**Fig. 5J**). Immunostaining for CD31 (endothelial marker) and laminin (vascular basement membrane) show vessels throughout the entire scaffold (**Fig. 5G, H**), with significantly greater vessel density (1.8-fold increase) and perivascular laminin deposition (1.5-fold increase) in scaffolds with acute TNF exposure (**Fig. 5J**). Quantitative RT-PCR analysis of these samples at the end of the 21-day induction period revealed significantly higher gene expression of VEGF-A (**Fig. 5I**). Additional gene expression markers are shown in **Figure S4**.

### *In vivo* integration of vascularized osteogenic grafts

**Figure 6:**
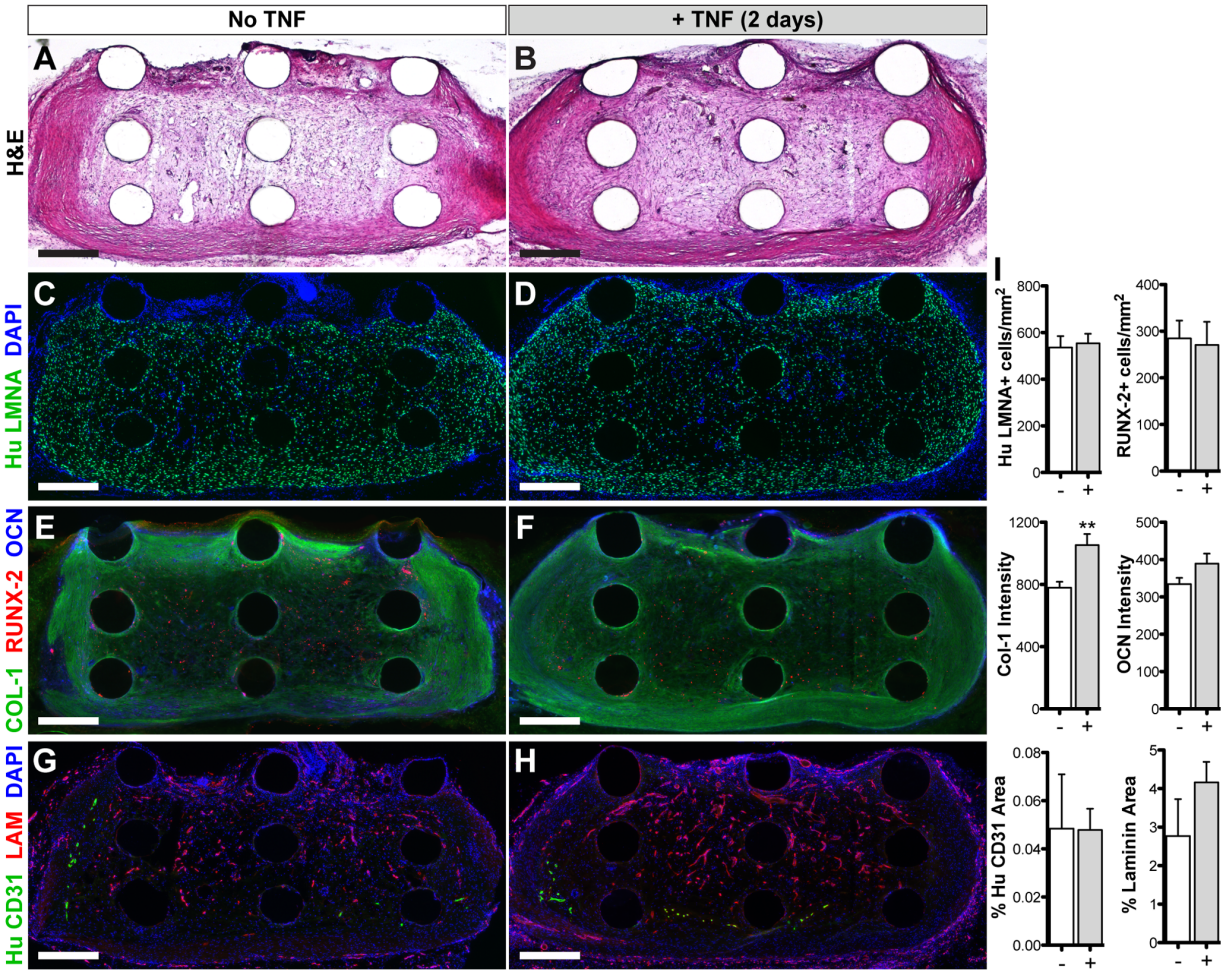
In vivo integration of vascularized osteogenic grafts. *In vitro*-induced grafts were implanted subcutaneously in athymic nude rats for 14 days. Staining of cryosections (10 µm thick): (**A, B**) H&E, (**C, D**) human-specific Lamin A/C (LMNA) to label implanted cells, (**E, F**) osteogenic markers, (**G, H**) vascular markers (human-specific CD31 and laminin (LAM) for rat/human vessels). Scale bars  = 500 µm. (**I**) Quantification of immunostaining. Values shown as mean ± SEM. Significance indicated as **p<0.01.

After 14 days *in vivo*, graft implants showed greater extracellular matrix production (**Fig. 6A, B**) than before implantation. The majority (55–65%) of the cells within the grafts stained positively for human-specific Lamin A/C (**Fig. 6C, D**), with no statistical difference in human cell density as a result of TNF exposure (**Fig. 6I**) nor when compared to the pre-implantation density (**Fig. 5J**), indicating high cell retention. Staining for bone matrix proteins collagen I and osteocalcin was significantly greater and more uniformly distributed compared to pre-implantation (4-fold and 3-fold change, respectively) and was also increased as a result of TNF treatment (**Fig. 6E, F, I**). The number of RUNX-2+ cells was similar amongst treatment groups, but significantly reduced compared to pre-implantation (0.5-fold change). Some human vessels remained viable within the grafts, although significantly less than what was implanted (0.2-fold change), with no significant difference amongst treatment groups (**Fig. 6G, H, I**). However, there was a dense infiltration of host vessels in all grafts (indicated by laminin staining), with 1.5-fold greater total vascular area in the TNF-treated group versus control.

## Discussion

Bone is a resilient tissue that has the potential to fully heal itself after moderate injuries. When this happens, normal healing progresses towards full recovery through a tightly regulated cascade of inflammation, cellular and vascular recruitment, and a complex host of signaling molecules. However in the case of large, critical-sized defects, these normal healing cascades are disrupted due to the shear size of the defect, as well as a number of confounding factors such as infection, mechanical instability, and absent sources of regenerative cues (i.e. periosteum, hematoma) [23], [24]. Historically, tissue engineers have vastly improved upon many of these shortcomings with the application of novel scaffolds, a variety of clinically relevant cells, and growth factors to instruct cells. More recently, inflammatory mediators have gained interest in the field because of their important roles in normal healing processes. The goal of this study was to study the effects of TNF, a key inflammatory cytokine, on vascular assembly and osteogenesis. We demonstrate that TNF acts synergistically with PDGF - another important component of the bone-healing cascade - to enhance vascularization within engineered osteogenic grafts.

Initial studies tested the effects of TNF dose on ASC osteogenesis and vascular morphogenesis in separate cultures, demonstrating that TNF has a beneficial effect on both lineages when supplied at a low dose for 48 hours (**Fig. 3**). While TNF induced a moderate increase in mineralization, this was concomitant with an increase in DNA content, suggesting that the increased mineralization may be due to proliferation. However, previous studies have indicated that TNF induces increased endogenous production of bone morphogenetic protein-2 (BMP-2) in mesenchymal stem cells [15], [16], suggesting that there may be a direct osteogenic effect on the cells. Not all pro-inflammatory cytokines elicit the same response, as we conducted a similar experiment with interleukin-1β (IL-1β) that resulted in a significant increase in mineralization for all concentrations ranging from 0.1–100 ng/mL, but a sharp dose-dependent inhibition of vascular assembly (**Fig. S5**).

In the *in vitro* model of vascularized bone development (**Fig. 4**), acute (2-day) treatment with TNF increased vessel length and interconnectivity. However, continuous exposure to the same TNF dose had an adverse effect on vascular network structure. This finding is supported by studies with endothelial cell cultures that have shown that continuous exposure to TNF can inhibit endothelial cell proliferation and growth factor responsiveness [8], [9]. In contrast to the monolayer osteogenesis experiment, mineralization was not significantly affected by the acute addition of TNF in the dual (vascular and osteogenic) induction model (**Fig. 4**). This is likely due to the fact that TNF was only added during the vascular phase of culture (first two days). With the addition of PDGF to these cultures, both vascularization and mineralization were improved, as was seen in our previous studies [1]. This PDGF-induced response was slightly synergistic in combination with the effects of TNF on the vascular development but not mineralization in these scaffolds. Ultimately, for in vivo applications, this improved vascular development is critical for graft survival and integration with host tissue.

Tissue development within composite fibrin-PCL scaffolds was studied before *in vivo* implantation because the spatial, mechanical, and biochemical transport properties are more complex than within a homogenous fibrin gel. Despite being uniformly seeded throughout the pores, the tissue formed more densely around the periphery of the scaffold (independent of TNF treatment) following three weeks of *in vitro* induction. This may be due to diffusion-limited gradients of oxygen and nutrients [25], given that cells form a layer of dense tissue on the scaffold periphery that may significantly reduce oxygen transfer to the central regions. Another hypothesis regards the differential constraints on the gel, which may remain fairly soft in the center, while the fibrin fibers near the edges where the PCL rods terminate are free to contract inward and stiffen [26]. Increased mechanical stiffness may play a role in facilitating the osteogenic response of ASCs [27]. Another observation at this time-point was that the majority of the cells in the central region of the scaffold expressed RUNX-2, an early marker of osteogenic differentiation, suggesting that these cells may be at an earlier stage of differentiation. Vascular growth within these scaffolds was significantly improved after TNF treatment, and may be due to an increase in endogenous VEGF-A expression. Interestingly, this increase in expression was detected at the end of the 3-week culture period, which was long after the TNF stimulus was removed, indicating a long-term effect on the cells.

After *in vivo* implantation, the pre-vascularized osteogenic grafts continued to mature, as indicated by the increase in H&E staining and greater deposition of collagen I and osteocalcin throughout the center of the graft. The decrease in RUNX-2+ cells (vs. pre-implantation) may indicate progression to a more mature phenotype. Interestingly, almost all of the implanted human cells were retained after 2 weeks *in vivo*. However, the amount of human vessels decreased significantly after implantation, showing no difference in retention as a result of TNF treatment. Previous studies have indicated that pre-engineered vascular networks may lead to faster anastomosis and subsequent remodeling and replacement by host vessels, suggesting that persistence of implanted vessels may not be necessary for a positive outcome [28]. Interestingly, host vascular invasion in this study was greater in the TNF-treated grafts, which may be due to improved remodeling and replacement of human vascular networks and/or elevated endogenous expression levels of VEGF-A and MMP-9 (**Fig. S4**), which are known to promote angiogenesis [29]–[31]. Therefore in the current study, acute TNF treatment had a greater long-term impact on vascularization.

An important consideration regarding these *in vivo* studies is the host inflammatory response. Nude Athymic rats were used for the animal model because they lack T-cells to enable xenograft implantation, but have normal B-cell and macrophage function [32]. In these animals, as well as immuno-competent animals or patients, there will undoubtedly be some level of innate immune response due to surgical implantation. On-going studies will investigate the role of this response on the development and functional integration of vascularized osteogenic grafts. From a clinical standpoint, the *in vivo* inflammatory environment can change based on the nature of the defect (e.g. an inflamed, compound injury versus congenital defect), infection, and anti-inflammatory drugs that the patient may be taking. While chronic inflammation and recurring infection have been shown to delay or inhibit healing of large bone defects [33], [34], the use of anti-inflammatory drugs have also been shown to slow healing rates [35]–[37]. These considerations further highlight the importance of understanding the role of the *in vivo* inflammatory environment in functional bone repair.

In summary, this study demonstrates how the pro-inflammatory cytokine TNF impacts vascularization of osteogenic grafts with ASCs. We have shown that acute exposure to TNF significantly improves vascular network formation within these grafts while prolonged exposure abrogates this response. The addition of exogenous PDGF synergistically enhances vascular development and simultaneously enhances mineral deposition. While this study demonstrates one application of *in vitro* pre-engineering of these grafts, the findings may have broader significance i.e. the approach may be applied to *in situ* tissue development strategies by controlling the exogenous or endogenous release of TNF *in vivo*. Our study underscores that modulation of the inflammatory response may be a powerful strategy to regulate tissue development during bone regeneration.

## Supporting Information

Figure S1 **Morphology of ASC aggregates after overnight suspension culture.** Scale bar  = 200 µm.
(TIF)

Figure S2 **Cylindrical polycaprolactone scaffold.** Dimensions: 4 mm diameter ×2 mm height, 350 µm average rod diameter, and 750 µm average pore width. Scale bar  = 500 µm.
(TIF)

Figure S3 **Flow cytometry double labeling for CD31 and PDGFR-β.** Passage 2 human ASCs were analyzed with flow cytometry for both CD31 and PDGFR-β, showing that all CD31+ cells are PDGFR-β+.
(TIF)

Figure S4 **RT-PCR analysis of TNF treatment.** Cells underwent dual (vascular and osteogenic) induction in the presence of 20 ng/mL PDGF (for 21 days) and either 0 or 0.1 ng/mL TNF (for 2 days), then assessed at day 21. Values shown as mean ± SEM. Significance indicated as *p<0.05 or **p<0.01 versus no TNF treatment.
(TIF)

Figure S5 **Effects of acute IL-1β exposure on independent lineage induction.** ASCs were induced towards either osteogenic differentiation (2D monolayer) or vascular morphogenesis (spheroids in 3D fibrin gel) and treated with varying doses of exogenous IL-1β for the first 48 hours. Osteogenic cultures were assessed via Alizarin Red S stain for calcium deposits (**A**), as well as quantification of total calcium content (**C**) and calcium normalized to DNA content (**D**) (dotted line: non-osteogenic control). Vascular cultures were assessed via whole-mount immunostaining for CD31 (green) and αSMA (red) (**B**), as well as quantification of vascular network length (**E**) and interconnectivity (**F**). Scale bars  = 500 µm. Values shown as mean ± SEM. *p<0.05, **p<0.01, or ***p<0.001 versus 0 ng/ml IL-1β.
(TIF)

Table S1 **Quantitative RT-PCR Primers.**
(DOCX)
