# Supplementary figures and images for: Tumor Necrosis Factor Improves Vascularization in Osteogenic Grafts Engineered with Human Adipose-Derived Stem/Stromal Cells
Source: PLoS One. 2014 Sep 23;9(9):e107199. doi: 10.1371/journal.pone.0107199 (PMC4172477; doi:10.1371/journal.pone.0107199)

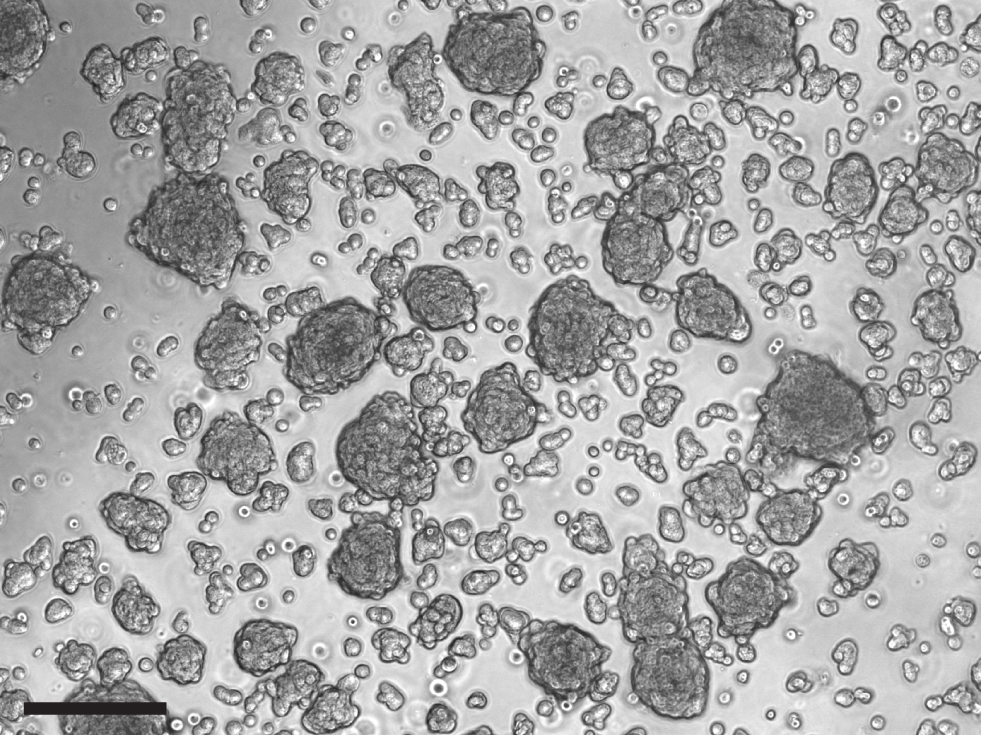

Supplement: Figure S1 — Morphology of ASC aggregates after overnight suspension culture. Scale bar = 200 µm. (TIF) [file pone.0107199.s001.tif]

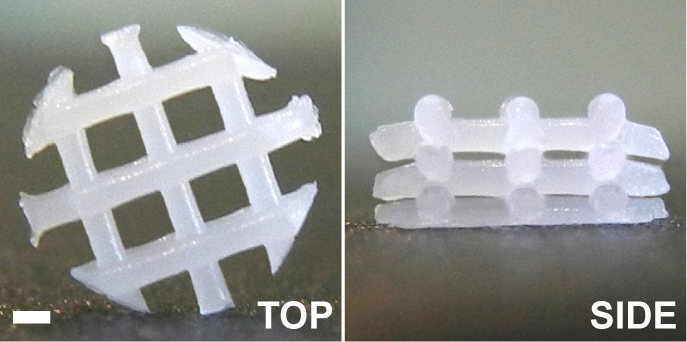

Supplement: Figure S2 — Cylindrical polycaprolactone scaffold. Dimensions: 4 mm diameter ×2 mm height, 350 µm average rod diameter, and 750 µm average pore width. Scale bar = 500 µm. (TIF) [file pone.0107199.s002.tif]

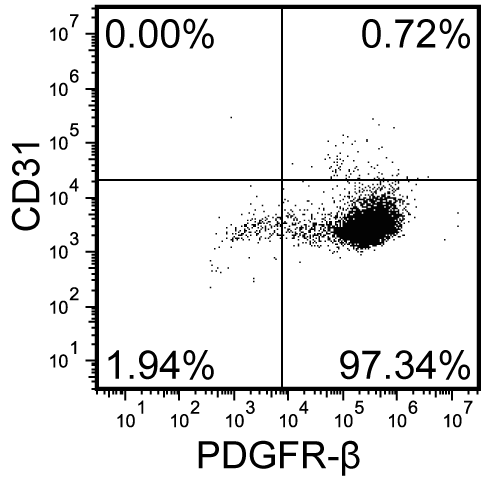

Supplement: Figure S3 — Flow cytometry double labeling for CD31 and PDGFR-β. Passage 2 human ASCs were analyzed with flow cytometry for both CD31 and PDGFR-β, showing that all CD31+ cells are PDGFR-β+. (TIF) [file pone.0107199.s003.tif]

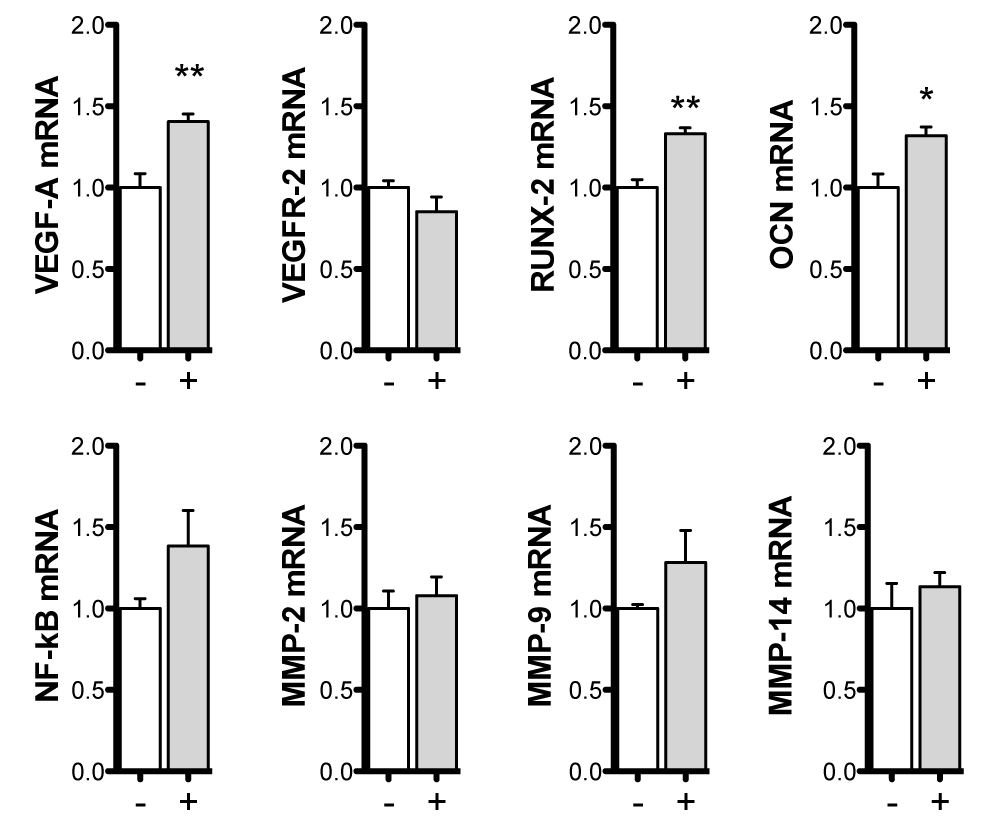

Supplement: Figure S4 — RT-PCR analysis of TNF treatment. Cells underwent dual (vascular and osteogenic) induction in the presence of 20 ng/mL PDGF (for 21 days) and either 0 or 0.1 ng/mL TNF (for 2 days), then assessed at day 21. Values shown as mean ± SEM. Significance indicated as *p<0.05 or **p<0.01 versus no TNF treatment. (TIF) [file pone.0107199.s004.tif]

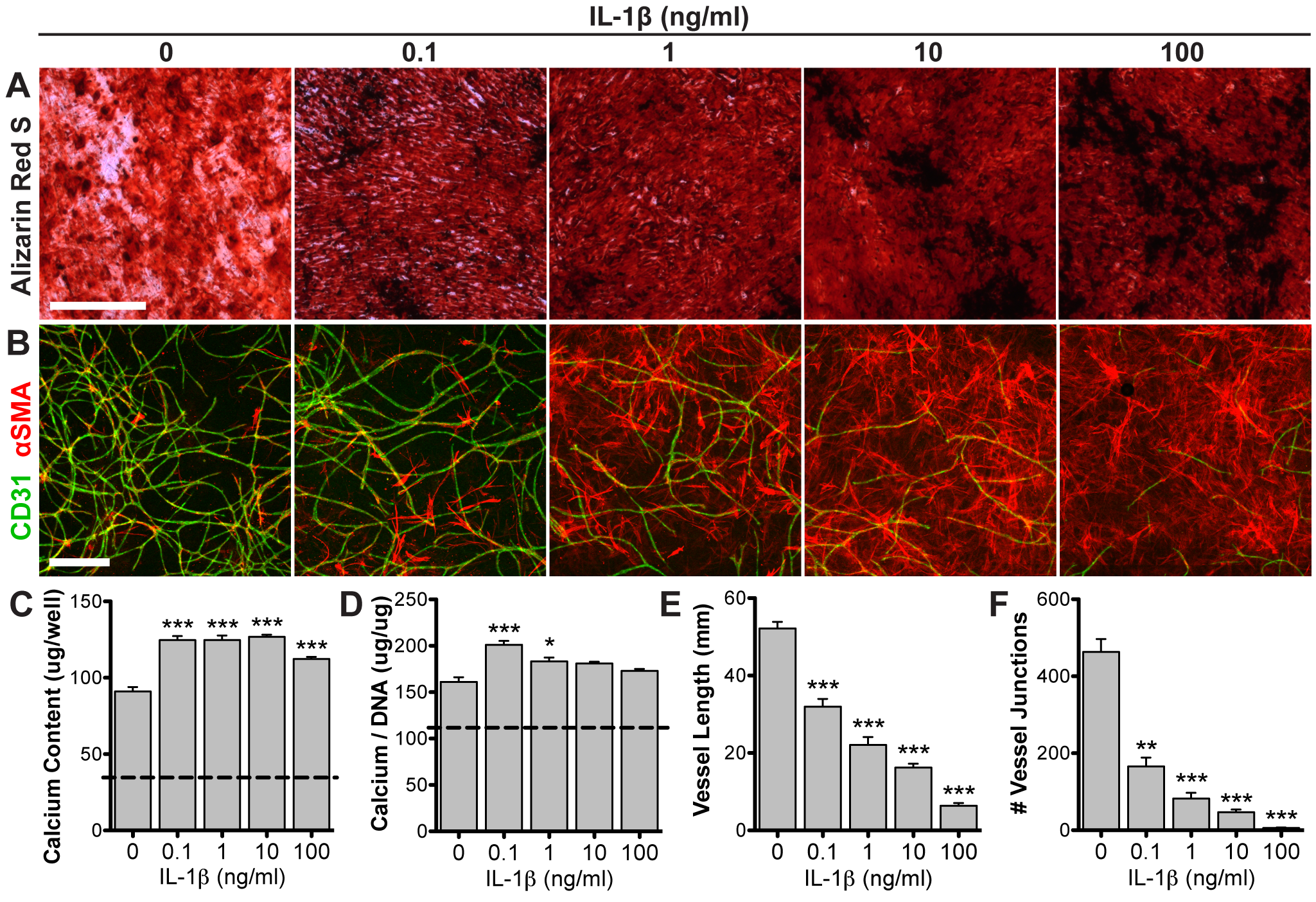

Supplement: Figure S5 — Effects of acute IL-1β exposure on independent lineage induction. ASCs were induced towards either osteogenic differentiation (2D monolayer) or vascular morphogenesis (spheroids in 3D fibrin gel) and treated with varying doses of exogenous IL-1β for the first 48 hours. Osteogenic cultures were assessed via Alizarin Red S stain for calcium deposits (A), as well as quantification of total calcium content (C) and calcium normalized to DNA content (D) (dotted line: non-osteogenic control). Vascular cultures were assessed via whole-mount immunostaining for CD31 (green) and αSMA (red) (B), as well as quantification of vascular network length (E) and interconnectivity (F). Scale bars = 500 µm. Values shown as mean ± SEM. *p<0.05, **p<0.01, or ***p<0.001 versus 0 ng/ml IL-1β. (TIF) [file pone.0107199.s005.tif]
