# Supplementary material for: Tumor Necrosis Factor Improves Vascularization in Osteogenic Grafts Engineered with Human Adipose-Derived Stem/Stromal Cells
Source: PLoS One. 2014 Sep 23;9(9):e107199. doi: 10.1371/journal.pone.0107199 (PMC4172477; doi:10.1371/journal.pone.0107199)
Supplement: Table S1 — Quantitative RT-PCR Primers. (DOCX) [file pone.0107199.s006.docx]

**Table S1:** Quantitative RT-PCR Primers

| **Gene Name** | **Primer Sequence (5’ – 3’)** | |
| --- | --- | --- |
| GAPDH | *Fwd.* | CACCCACTCCTCCACCTTTGA |
|  | *Rev.* | TCCACCACCCTGTTGCTGTAG |
| VEGF-A | *Fwd.* | GCCTTGCCTTGCTGCTCTA |
|  | *Rev.* | GATTCTGCCCTCCTCCTTCTG |
| VEGFR-2 (KDR) | *Fwd.* | AGTCTGTGGCATCTGAAGGC |
|  | *Rev.* | ACGGTGGTGTCTGTGTCATC |
| RUNX-2 | *Fwd.* | GTCTCACTGCCTCTCACTTG |
|  | *Rev.* | CACACATCTCCTCCCTTCTG |
| NF-κB | *Fwd.* | TGTGGTGGAGGATTTGCTGAG |
|  | *Rev.* | TGGTCAAGAAGTAGTGCTGCC |
| MMP-2 | *Fwd.* | AGCATTCTCACTCCTACC |
|  | *Rev.* | CAGTCAGCATCTATTCTTGG |
| MMP-9 | *Fwd.* | GGGCAGATTCCAAACCTT |
|  | *Rev.* | GGCAAGTCTTCCGAGTAG |
| MMP-14 | *Fwd.* | CAGGCACTTTGAGGAACA |
|  | *Rev.* | TCGGTAGGCACTGAACTT |
